# Implementation of MRSA Nasal Swabs as an Antimicrobial Stewardship Intervention to Decrease Anti-MRSA Therapy in COVID-19 Infection

**DOI:** 10.3390/antibiotics12020253

**Published:** 2023-01-27

**Authors:** Alaina DeKerlegand, Emily Johnston, Britney Mellor, Melanie Rae Schrack, Catherine O’Neal

**Affiliations:** 1 Pharmacy Department, Methodist University Hospital, Memphis, TN 38104, USA; 2 Pharmacy Department, Our Lady of the Lake Regional Medical Center, Baton Rouge, LA 70808, USA; 3 Medical Staff Office, Our Lady of the Lake Regional Medical Center, Baton Rouge, LA 70808, USA; 4 Louisiana State University Health Sciences Center, Baton Rouge, LA 70808, USA

**Keywords:** COVID-19, SARS-CoV-2, MRSA nasal swabs, MRSA pneumonia, antimicrobial stewardship

## Abstract

In the early stages of treating patients with SARS-CoV-2, limited information was available to guide antimicrobial stewardship interventions. The COVID-19 Task Force and Antimicrobial Stewardship Committee, at a 988-bed academic medical center, implemented the use of methicillin-resistant *Staphylococcus aureus* (MRSA) nasal swab polymerase chain reaction (PCR) testing to assist with the de-escalation of anti-MRSA therapy in patients with suspected superimposed bacterial pneumonia in COVID-19. A retrospective study was conducted to evaluate the impact of MRSA nasal swab PCR testing on the rate of anti-MRSA therapy between 13 April 2020 and 26 July 2020. A total of 122 patients were included in the analysis. Of the patients included in the final analysis, 58 (47.5%) had anti-MRSA therapy discontinued and 41 (33.6%) avoided anti-MRSA therapy completely due to a negative swab result. With the implementation of MRSA nasal swab PCR testing in COVID-19 patients, anti-MRSA therapy was reduced in 81% of patients in this study. In patients who continued with anti-MRSA therapy, nasal swabs were either positive for MRSA or an alternative indication for anti-MRSA therapy was noted. Only three patients in the cohort had MRSA identified in a sputum culture, all of whom had anti-MRSA therapy continued. MRSA nasal swab PCR testing may serve as an effective antimicrobial stewardship tool in COVID-19 pneumonia.

## 1. Introduction

With the rapid emergence of SARS-CoV-2 in 2019, researchers and clinicians worked quickly to understand the virus and its associated clinical presentation, COVID-19, to optimize patient management. One initial area of concern was the unknown risk of bacterial co-infections and how to guide empiric antibiotic therapy. Patients with influenza experience rates of bacterial coinfections at 11–35% based on the reported literature, with *Staphylococcus aureus* (*S. aureus*) representing 28% of reported pathogens in one meta-analysis [1,2]. Although there is no recommendation for the addition of coverage for methicillin-resistant *S. aureus* (MRSA) post-influenza in guidelines for community-acquired pneumonia (CAP), many clinicians opt to add coverage based on the incidence seen in prior studies [3]. Due to the high mortality rate seen in COVID-19, many additional patients would meet the criteria for empiric coverage of MRSA in hospital-acquired pneumonia (HAP) and ventilator-associated pneumonia (VAP) as well [4]. An early meta-analysis reviewed the literature on known coronavirus strains and rates of bacterial co-infection; *S. aureus* has been isolated in patients with prior coronavirus infections at a rate as high as 47.1%, which was found in one cohort of patients who were admitted to the ICU with ventilator-associated pneumonia from SARS-CoV-1 [5]. Among the studies on SARS-CoV-2 included in this analysis, an overall bacterial or fungal co-infection rate of 8% was observed [5]. Although five studies did not report the specific organisms identified, there was only one patient with (unspecified) gram-positive organisms reported in the cohort [5]. Subsequently, Lansbury and colleagues reported a similar bacterial co-infection rate of 7%, with one reported case of MRSA [6]. This provided promising preliminary data that *S. aureus* co-infections may be rare events in patients with COVID-19. However, the initial guidelines for COVID-19 management from the Infectious Diseases Society of America (IDSA) did not provide recommendations regarding suspected bacterial pneumonia or antibiotic coverage [7]. Uncertainty among providers on the front lines led to a continuation of the increased use of empiric antibiotics.

Due to concerns of *S. aureus* bacterial pneumonia in COVID-19, as well as the hospital rate of methicillin-resistance of 66% for *S. aureus*, anti-MRSA therapy was added to many empiric regimens at this facility. To decrease antibiotic utilization, minimize cumulative acute kidney infection risk, and limit nursing exposure from additional drug administrations and drug level monitoring, the antibiotic stewardship committee implemented routine MRSA nasal swab polymerase chain reaction (PCR) testing in all COVID-19 patients, suspected or confirmed, receiving anti-MRSA therapy. Studies published to date have supported the use of MRSA nasal swab PCR screening as a tool for deescalating anti-MRSA coverage in pneumonia, with reports of negative predictive values >96% in most studies [8,9,10,11,12,13]. However, the efficacy in patients with COVID-19 infections has yet to be established. The goal of this study is to evaluate the impact of the use of MRSA nasal swab PCR screenings in patients with COVID-19 pneumonia on the duration of anti-MRSA antibiotic therapy.

## 2. Materials and Methods

### 2.1. Study Overview

A retrospective review was conducted to evaluate the use of MRSA nasal swabs in patients admitted to Our Lady of the Lake Regional Medical Center between 13 April 2020 and 26 July 2020. Electronic Health Record (Epic)-generated reports were created to identify patients with a diagnosis code of COVID-19 infection or COVID-19 rule-out who had also received an MRSA PCR nasal swab. This study was approved through the Institutional Review Board (IRB) at both Franciscan Missionaries of Our Lady University and the Louisiana State University Health Science Center. Patients were excluded from analysis if the MRSA nasal swab and SARS-CoV-2 PCR were ordered only as pre-operative rule-out measures or if they were treated at other hospital sites within the health system.

### 2.2. Intervention

**Figure 1:**
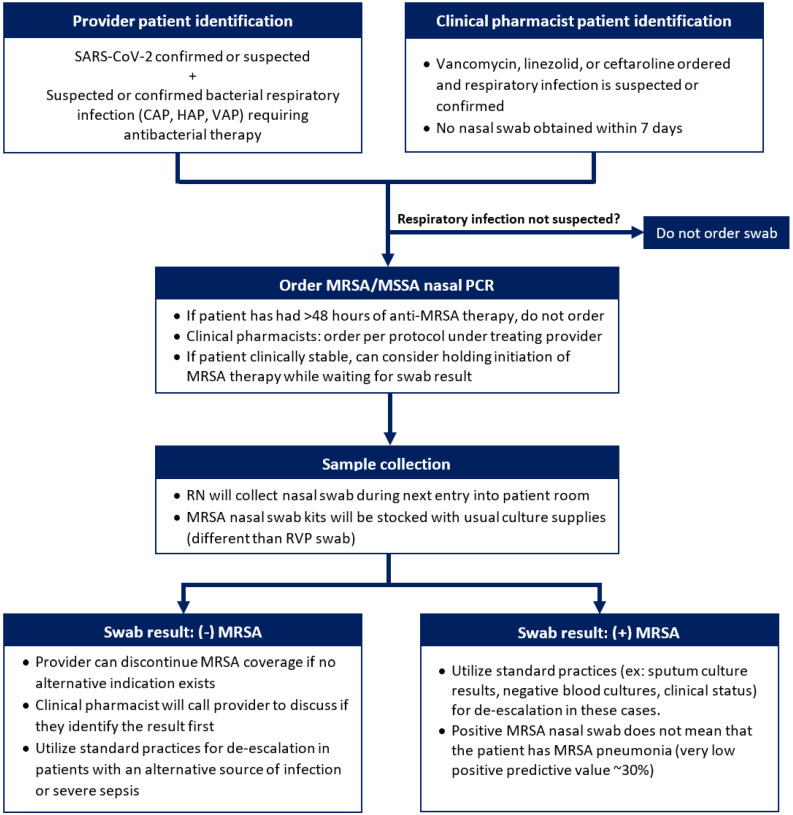
Workflow for ordering, obtaining, and interpreting the results of the MRSA PCR nasal swabs.

Due to the rapidly escalating number of admitted COVID-19 patients in April 2020, a protocol for MRSA therapy de-escalation was reviewed by the antimicrobial stewardship committee. It was then provided to physicians, nurses, and clinical pharmacists with information on the rationale and workflow for ordering, obtaining, and interpreting the results of the MRSA PCR nasal swabs (see Figure 1). Red Copan nasal swabs were utilized for sample collection and analyzed through the Xpert SA Nasal Complete assay. Direct education also occurred in physician and pharmacist departmental meetings and on multidisciplinary rounds. Physicians were instructed to order an MRSA PCR nasal swab on patients with confirmed or suspected SARS-CoV-2 as well as suspected or confirmed bacterial pneumonia requiring antibacterial therapy; clinical pharmacists could order the swab on any applicable patients initiated on vancomycin, ceftaroline, or linezolid without a prior swab order. If the nasal swab resulted as negative for MRSA, and the patient’s only indication for anti-MRSA therapy was pneumonia, recommendations were made to the provider from the pharmacy team to discontinue anti-MRSA antimicrobial therapy. To ensure the accuracy of the swab collections, nursing education was created with assistance from the manufacturer’s recommendations and input from nursing educators. Education was initially provided to nursing staff by an infectious diseases clinical pharmacist, and this was repeated by nursing directors during staff huddles. Education sheets were also posted on all units with cohorted COVID-19 patients as a reference for nursing staff.

### 2.3. Data Collection and Analysis

Baseline demographic information and data on the severity of the illnesses, including ICU admission and ventilator status, was collected. Mortality data were obtained from the electronic health records. All culture data during the patient’s admission was reviewed to evaluate the rate of bacterial co-infection with COVID-19 among this patient population. Organisms typically representative of contamination, such as coagulase-negative *Staphylococcus* identified in a single blood culture, were not included as cases of infection. Additionally, instances of colonization were not included. Physician notes were reviewed for any cases of uncertainty. The primary objective was analyzed through collection of the MRSA PCR nasal swab results, duration of anti-MRSA therapy, and review of provider notes when necessary to confirm provider decisions. Patients were grouped based on the provider’s decision to discontinue or continue anti-MRSA therapy based upon the results of the MRSA PCR nasal swab. A power analysis was not performed, and results were analyzed with descriptive statistics.

## 3. Results

The electronic health record-generated report identified a total of 13,647 patients with a COVID-19 infection or COVID-19 rule-out orders in the hospital system during the study period, 353 of whom had also received an MRSA PCR nasal swab. A total of 231 patients were excluded due to swabs being perioperative orders or due to having received treatment at other hospitals within the system, leaving 122 patients included in the final analysis (62% male, median age 65 years). The full baseline demographics are shown in Table 1.

In this cohort, 84.4% of patients were diagnosed with COVID-19 based upon SARS-CoV-2 PCR results. It is important to note that due to difficulties with testing at this early stage of the COVID-19 pandemic, some patients may have had false negative results. Approximately 75% of all patients in this study required ICU admission, with 61 (50% overall, 66% of ICU patients) requiring mechanical ventilation during their admission. The overall mortality rate was 43.4%. There was a trend toward higher rates of definitive COVID-19 diagnosis, ICU admission, ventilator requirement, and mortality in patients in the continued arm.

Positive blood and/or sputum cultures were seen in 24 (20%) patients in this study. The median time from admission to positive culture was 6 days. A total of 9 cultures were positive within 48 h of admission, indicative of community-acquired infections; the remaining 15 were collected more than 48 h after admission, and represented potential hospital-acquired infections. Respiratory cultures were obtained in a total of 35 patients, with 16 overall patients (13.1%) having at least one organism identified. *Staphylococcus aureus* was isolated in 3 of 35 cultures (8.6%). *S. aureus* accounts for 15.8% of the total organisms isolated in the respiratory tract, and all *S. aureus* isolates were methicillin-resistant. Overall, MRSA was isolated in the cultures of 2.5% of patients in the study. One of the three patients also developed MRSA bacteremia. The mortality rate was higher, at 58% among the 35 patients with positive blood and/or sputum cultures, and at 100% for the 3 patients with MRSA identified in sputum cultures.

**Figure 2:**
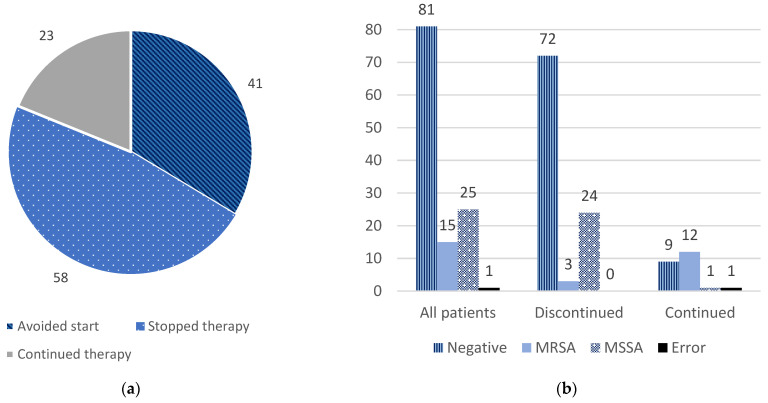
MRSA PCR nasal swab results and responses. (**a**) Provider responses to swab results. Avoided start and stopped therapy collectively form the group of discontinued therapy. (**b**) Provider responses, stratified by result of MRSA PCR nasal swab.

MRSA PCR nasal swabs were ordered a median of 2 days after admission. A total of 15 swabs overall (12.3%) resulted as positive for MRSA. In response to the swab results, 58 patients (47.5%) had anti-MRSA agents discontinued and another 41 patients (33.6%) were never started on anti-MRSA therapy (see Figure 2a). Patients with anti-MRSA therapy which was either discontinued or never started collectively are referred to herein as “discontinued” for ease of analysis (see Table 1 and Figure 2a). Of the 12 patients with therapy continued, 6 had MRSA positive swabs, 1 had a swab error, and 3 had therapy continued due to sepsis of an unknown origin (See Figure 2b). When considering the 83 patients in the study who received anti-MRSA therapy, the median duration overall was 48 h (IQR 24, 96). The median duration differed between groups, with a median of 24 h (IQR 12, 60) in the 60 patients receiving anti-MRSA therapy in the discontinued group versus 120 h (IQR 96, 168) in the continued group. A secondary analysis was performed to additionally include the 39 patients in the discontinued group who did not receive anti-MRSA therapy as a duration of therapy of 0 h; the median duration of therapy overall in the study population would be 24 h (IQR 0, 72).

## 4. Discussion

During the early stages of the COVID-19 pandemic, limited information was available regarding co-infections to guide antibiotic therapy [5,6]. Additional data have continued to emerge which further elucidate the rate of bacterial co-infections in patients with COVID-19. One large multi-site review, which included 3412 patients, found an incidence of bacterial co-infection of 3.5% [14]. An observational cohort study identified a rate of 3.1% for community-acquired bacterial co-infections and 4.4% for hospital-acquired bacterial co-infections [15]. One large meta-analysis (including 75,956 evaluable patients) identified an overall rate of bacterial coinfections of 4.7%. *S. aureus* was the most frequently reported pathogen among the studies included in this meta-analysis [16]. A total of 24 patients (20%) in this study were found to have positive blood and/or sputum cultures. However, this rate is not directly comparable to the aforementioned studies, as this population focused on those with suspected bacterial co-infection. Ultimately, the incidence of concomitant bacterial infection in any patient may depend on multiple factors, which include baseline immunocompromising conditions, SARS-CoV-2 variant, and immunosuppressive therapies, including high-dose steroids and baricitinib. Updates to the IDSA guidelines on COVID-19 management note the generally low rates based on the available literature [17]. Although the incidence of bacterial infections in patients with COVID-19 appears to be relatively low, the rate of resistant infections may be on the rise. The Centers for Disease Control (CDC) released a special report in 2022 to describe the impact of COVID-19 on antimicrobial resistance in the US, which noted that despite previous stabilization of rates of MRSA infections, the overall rate began to increase again in 2020 [18]. Although these infections were not attributed to patients with COVID-19 specifically, it possible that the overall incidence was impacted by the COVID-19 pandemic.

Without clear guidance, antimicrobial stewardship during the pandemic has remained a concern and a priority. In one large multi-site retrospective review of adult inpatients with COVID-19, over 50% of patients had received empiric antibiotics; in 15% of overall study patients, the antibiotic regimens included anti-MRSA therapy [14]. In a meta-analysis of adult inpatients with COVID-19, over 62.4% of patients received empiric antibiotics, with vancomycin representing 6.1% of antibiotics prescribed [19]. Alshaikh and colleagues found antibiotic use in 73% of patients despite the confirmed rate of bacterial coinfection of only 4.7% among patients in the large meta-analysis [16]. Amidst the difficulties surrounding antimicrobial stewardship, Staub and colleagues were able to demonstrate a reduction in the duration of antibiotic therapy in COVID-19 patients with the implementation of institutional guidance [20]. The available literature on the management of patients with COVID-19 indicates a strong need for evidence of effective antimicrobial stewardship strategies.

The complex disease process of COVID-19 also led to difficulties with antibiotic selection. The early literature on patients with COVID-19 noted that there were high rates of AKI, ranging from 3.2 to 25.5% [21,22,23]. This may be due to a combination of factors, including, but not limited to, sepsis, vasopressor requirements, coagulopathies, or direct SARS-CoV-2 activity in the kidneys [24,25]. One study characterizing the mortality of patients with COVID-19 admitted to the intensive care unit (ICU) showed that 29% of the 217 patients included required some form of dialysis during their admission, and that the mortality rate for patients requiring dialysis was 44.5% (compared to an overall mortality rate of 25.8%) [22]. It became critical to limit exposure to nephrotoxic agents in order to reduce the overall risk. The rate of AKI associated with vancomycin use varies widely among the reported literature and remains a clinical controversy. The incidence may depend upon vancomycin area under the curve (AUC) exposure with each patient [26,27]. Critical illness is considered to be a factor that increases the risk of developing AKI while receiving vancomycin [28]. Medication shortages, drug interactions, and risk of thrombocytopenia limited the utility of linezolid as well [29,30].

In this study population, empiric coverage for MRSA was reasonable based on the elevated mortality rate and the hospital methicillin-resistance rate of 66% in *S. aureus* isolates [3,4]. Although empiric coverage may have been warranted, guidelines support de-escalation based on cultures or other data without any negative impact on mortality or length of hospital stay. Prior studies have shown that MRSA nasal PCR screening is a useful tool for de-escalation of anti-MRSA therapy in bacterial pneumonia treatment [8,9,10,11,12,13]. At this large academic medical center, MRSA PCR nasal swabs were historically used for inpatients requiring surgical procedures. Based upon de-identified data from the microbiology lab, the positivity rate for MRSA in nasal swabs in 2019 was 8%, and *Staphylococcus aureus* (MRSA and MSSA collectively) was identified in 9% of all respiratory cultures obtained that year. The results of this study are similar to historic data, with a rate of MRSA in 12.3% of nasal swabs. Three patients in this study had *Staphylococcus aureus* (methicillin-resistant), identified in sputum cultures (2.4% of patients). Notably, only 35 patients in this study had a sputum culture obtained during their treatment course, with gram-negative organisms isolated in 13 cultures. No patients in the discontinued group developed subsequent MRSA infections during their hospital stay. With the implementation of MRSA nasal swabs in COVID-19 patients, we were able to reduce or avoid anti-MRSA therapy in 81% of patients in the study cohort, with a median duration of anti-MRSA therapy of 24 h in the discontinued group (or 12 h in the subgroup analysis, which included the 39 patients in the discontinued group who avoided anti-MRSA therapy, indicated by a duration of therapy of 0 h).

There are several limitations of this retrospective review. The number of patients who qualified for an MRSA PCR nasal swab but received usual care only is unknown. However, a small group of clinical pharmacists were performing clinical profile reviews and vancomycin dosing for patients with COVID-19 during this time frame. Since all were given education on the process and frequent reminders to order MRSA PCR nasal swabs for qualifying patients, it is likely that most qualifying patients had the appropriate MRSA PCR nasal swabs ordered. At this time, we were also unable to compare the duration of anti-MRSA therapy before and after the protocol was introduced. Due to rapid protocol implementation and applicability to all patients with COVID-19, it is anticipated that limited patients would be available as a comparator group. In addition, a lower rate of respiratory culture collection may be present in COVID-19 patients due to perceived exposure risk and PPE shortages as compared to other populations. This may limit our estimation of the true prevalence of bacterial pneumonia with COVID-19 infection. In our cohort of COVID-19-infected patients who received an MRSA nasal swab for suspected bacterial pneumonia, only 29% had a sputum culture collected. An additional limitation of this study is in relation to the determination of provider responses to nasal swab results. In all patients in the cohort, it was possible to determine that all providers wanted anti-MRSA therapy for their patients based on antibiotic orders and/or statements made in notes. However, a small number of providers opted to delay initiation by an hour while awaiting nasal swab results. For patients who received a nasal swab but never had an order for an anti-MRSA antibiotic, it was possible to determine from the notes that the provider elected against initiating therapy based on the negative swab result. However, based on varying time points in relation to nasal swabs (e.g., time of swab order, time of swab collection, time of swab results) and unknown time points (e.g., time that the provider saw the nasal swab result), it is not possible to definitively determine whether the initiation of anti-MRSA therapy was based on the result of a nasal swab. However, only 1 patient of the total 22 in the continued group had anti-MRSA therapy ordered after the nasal swab result became available. Lastly, this study only included a review of patients during the initial COVID-19 wave with the wild-type virus, and direct extrapolation to SARS-CoV-2 variants cannot be confirmed through this study.

Results of this single-center, retrospective review indicate positive results for the use of MRSA nasal PCR screening for de-escalation of anti-MRSA therapy in patients with COVID-19 pneumonia. Based upon the positive results of this preliminary study, the MRSA nasal swab protocol for COVID-19 patients was continued. Physicians’ responses to the protocol’s implementation have been positive to date, given most swabs were physician-ordered (57%) and the rate of medication discontinuation based on the swab result was high (81%). Although not recommended as part of the protocol, many physicians elected to order an MRSA nasal PCR and await results prior to determining whether to start anti-MRSA therapy; this resulted in complete avoidance of anti-MRSA therapy in 33.6% of the study population. Based upon the high acceptance rate to discontinue or avoid therapy, as well as over half of orders being placed by physicians, there appeared to be rapid acceptance of the protocol. Based on this rapid acceptance of the protocol, it was later expanded to include all patients receiving anti-MRSA therapy as part of their empiric pneumonia treatment. Although not quantified in this preliminary review, it is anticipated that this reduction in anti-MRSA therapy assisted with decreasing PPE usage through limiting entries for drug administration and lab draws. It is also plausible that a reduction in vancomycin exposure could reduce the rate or severity of AKI development in this critically-ill cohort.

To our knowledge, this is the first study which has evaluated the use of MRSA nasal PCR screening specifically in patients with suspected bacterial pneumonia secondary to COVID-19. Based on the results of this study, the MRSA nasal PCR screening did not yield any false negative results and could be expected to retain a high negative predictive value in this specific patient population. Follow-up studies which include patients with SARS-CoV2 variants are warranted to further evaluate the utility of this protocol in COVID-19 pneumonia.

## Figures and Tables

**Table 1:** Baseline demographics and results.

	All		Discontinued		Continued	
	N = 122	%, IQR	N = 99	%, IQR	N = 23	%, IQR
Male	76	(62.3)	63	(63.6)	13	(56.5)
Age *	65	(56, 72)	65	(55, 72)	65	(57, 72)
COVID-19 diagnosis	103	(84.4)	81	(81.8)	22	(95.7)
Length of stay * (days)	12	(6, 19)	11	(5, 20)	11	(6, 19)
ICU admission	92	(75.4)	73	(73.7)	19	(82.6)
ICU LOS * (days)	12	(7, 17)	12	(6, 18)	9	(6, 13)
Admit to ICU * (days)	1	(0, 3)	1	(0, 3)	1	(0, 3)
Ventilated	61	(50.0)	46	(46.5)	15	(68.2)
Mortality	53	(43.4)	42	(42.4)	11	(47.8)
Time from admit to swab * (days)	2	(1, 7)	2	(1, 6)	2	(1, 9)
Swab ordering user						
Physician	70	(57.3)	61	(61.6)	9	(39.1)
Pharmacist	45	(36.9)	32	(32.3)	13	(56.5)
Nurse	7	(5.7)	6	(6.1)	1	(4.3)
MRSA agent						
Vancomycin	50	(41.0)	34	(34.3)	16	(70.0)
Linezolid	33	(27.0)	27	(27.3)	6	(26.1)
Ceftaroline	4	(3.3)	3	(3.0)	1	(4.3)
None	35	(28.7)	35	(35.4)	0	(0.0)
Positive blood cultures	13	(10.7)	11	(11.1)	2	(8.7)
Time to positive * (days)	3	(0, 12)	8	(0, 12)	0	(0, 0)
Sputum culture collected	35	(28.7)	26	(26.2)	9	(39.1)
Sputum pathogen #	16	(13.1)	10	(10.1)	6	(26.1)
Time to positive * (days)	9	(6, 13)	10	(6, 17)	6	(2, 10)

* Reported as median and interquartile range. # Sputum pathogens isolated in 16 total cultures, 3 of which had 2 pathogens isolated. Pathogens in culture included *Pseudomonas aeruginosa* (9 cultures), Enterobacterales (6 cultures), MRSA (3 cultures), and *Moraxella catarrhalis.*

## Data Availability

Not applicable.
